# Supplementary material for: Severe Lymphatic Disorder and Multifocal Atrial Tachycardia Treated with Trametinib in a Patient with Noonan Syndrome and SOS1 Mutation
Source: Genes (Basel). 2022 Aug 23;13(9):1503. doi: 10.3390/genes13091503 (PMC9498305; doi:10.3390/genes13091503)
Supplement: Supplementary file 1 [file genes-13-01503-s001.zip › genes-1850807-supplementary.pdf]

Supplementary Materials

Table S1. Proposed screening protocol, including ophthalmological, cardiological, dermatological, radiographic and hematological evaluation, performed to monitor for possible side effects based on clinical trials.

Exam	B	1w	2w	3w	4w	5w	6w	7w	8w	9w	10w	11w	12w	13w	14w	16w
Cardiovascular evaluation	X	X			X	X				X	X		X	X	X	X
ECG	X	X	X	X	X	X	X	X	X	X	X	X	X	X	X	X
Echocardiogram	X	X			X		X		X		X		X		X	X
24h Holter monitoring	X	X	X		X		X		X		X		X		X	X
Serum troponin/ NT-proBNP	X	X	X		X		X		X		X		X			X
Chest CT scan	X				X				X				X			X
Ultrasonography: abdomen	X				X				X				X			X
Ultrasonography: lung	X	X		X				X				X				X
Dermatological evaluation			X						X					X		
Biochemical/ full cell count[a]	X	X		X	X		X		X				X			X
Ophtalmological evaluation			X										X			

B: baseline treatment initiation w: week a: haemocrome, serum creatinine, electrolytes, C-reactive protein, procalcitonin, pro BNP, thyroid hormones, transaminase enzymes.
